# Ultrasound Evaluation of the Abductor Hallucis Muscle in Youth: A Reliability Study

**DOI:** 10.1002/jfa2.70230

**Published:** 2026-10-29

**Authors:** Maisie Squibb, Dean Monks, Christopher D. Nulty, Kelly Sheerin, Peter Francis

**Affiliations:** ^1^ Department of Health & Sports Science The EVOLVE Research Group South East Technological University (SETU) Carlow Ireland; ^2^ Sport Research Institute New Zealand (SPRINZ) School of Sport, Exercise and Health Auckland University of Technology Auckland New Zealand

**Keywords:** anatomy, children, foot structures, medial longitudinal arch, paediatrics

## Abstract

**Introduction:**

Abductor hallucis (AbdH) is an intrinsic foot muscle and a stabiliser of the medial longitudinal arch. Its cross‐sectional area (CSA) is routinely measured by ultrasound (US) and is highly relevant to foot muscle development. However, the reliability of portable US for measuring AbdH CSA in healthy youth is unknown. Therefore, the aim of this study was to examine the inter‐device association, inter‐rater reliability and inter‐session reliability of portable US for measuring AbdH CSA in youth.

**Methods:**

Twenty‐seven youths (11–17 years; 14 male, 13 female) participated, with inter‐device association and inter‐rater reliability assessed in a subgroup of *n* = 12 and intra‐rater inter‐session reliability in *n* = 15. AbdH CSA images were captured on two occasions one week apart using a 5–15 MHz handheld US unit (L15HD, Clarius), while a single image was captured using a cart‐based US device (3–16 MHz; HS40, Samsung) for inter‐device comparison. Reliability was assessed using intraclass correlation coefficients (two‐way mixed‐effects, absolute agreement). Inter‐device comparison was evaluated using Pearson's correlation, linear regression and paired‐sample *t*‐test for systematic differences, and measurement error was quantified using the standard error of measurement (SEM).

**Results:**

Portable and cart‐based US measurements of AbdH CSA showed a strong positive correlation (*r* = 0.938, *p* < 0.001), although mean values were 0.19 cm^2^ higher with the cart‐based device (*p* = 0.002). Inter‐rater reliability was excellent (ICC(3, 3) = 0.934, 95% CI: 0.786–0.980) with no significant difference between assessors (*p* = 0.823), minimal bias (−0.01 cm^2^), and a SEM of 0.039 cm^2^. Inter‐session reliability was also excellent (ICC(3, 3) = 0.969, 95% CI: 0.911–0.989) with no significant difference between sessions (*p* = 0.788), minimal bias (0.007 cm^2^) and a SEM of 0.013 cm^2^.

**Conclusion:**

A portable US device demonstrated excellent inter‐rater and inter‐session reliability for measuring AbdH CSA in youth and was strongly associated with cart‐based US measurements.

**Abbreviations**
AbdH: abductor hallucis
CSA: cross‐sectional area
ICC: intra‐class correlation coefficient
IFM: intrinsic foot muscles
SEM: Standard error of measurement
US: ultrasound

## 1 Introduction

The intrinsic foot muscles (IFM), comprising 29 muscles organised into four layers, function as a critical unit that stabilises and supports the medial longitudinal arch [1, 2, 3]. IFM are active during functional movements and contribute to foot stabilisation and support of the medial longitudinal arch, while IFM weakness has been associated with pes planus (flat feet) and excessive pronation [4]. Abductor hallucis (AbdH) is the most medial superficial IFM and plays a vital role as a dynamic stabiliser. Weakness in the AbdH is frequently linked to common foot deformities, such as hallux valgus and pes planus [5, 6, 7, 8]. Improving AbdH strength may minimise deformity and enhance functional movement in adults [9, 10, 11]; however, evaluating individual IFM strength is difficult because these muscles are small and share similar functions [12]. Consequently, measurement of muscle cross‐sectional area (CSA) is used as an indirect indicator of IFM strength [4]. This approach allows assessors to evaluate individual muscles [4, 13, 14], given the known moderate‐to‐strong correlation between muscle CSA and force production [13, 14]. However, during childhood and adolescence, the foot continues to undergo structural and functional maturation [15, 16]. The IFM has not yet fully matured or reached peak mass or strength [15], and smaller muscle size may affect an assessor's ability to obtain clear images of the AbdH.

Ultrasound (US) provides a low‐cost, non‐invasive, and accessible method for assessing IFM morphology and has demonstrated high inter‐rater reliability and comparable measurements to MRI for the measurement of AbdH, flexor hallucis brevis, flexor digitorum brevis, quadratus plantae, and abductor digiti minimi in young to middle‐aged adults [17]. Recent improvements to affordable, portable US devices with higher resolution have enabled more research into IFM morphology [18, 19, 20, 21], and the portability of these small transducers could allow measurement of smaller muscles in previously challenging environments, such as school settings. Evidence from a small number of studies indicates that portable US devices can reliably assess AbdH CSA in adults and older adults, but reliability data in youth populations remain lacking [18, 22, 23]. Therefore, the aim of this study was to investigate the inter‐device association, inter‐rater reliability, and inter‐session reliability of portable US for measuring AbdH CSA in youth. Previous studies have reported good‐to‐excellent reliability of portable US for measuring AbdH CSA in adults and older adults [18, 22, 23], suggesting that similar reliability may be achievable in younger populations. It was hypothesised that portable US would demonstrate high inter‐rater and inter‐session reliability for measuring AbdH CSA in youth and a strong association with measurements obtained using cart‐based US.

## 2 Methods

### 2.1 Study Design and Participants

This study examined the inter‐device association, inter‐rater reliability, and intra‐rater inter‐session reliability of portable US for measuring the CSA of the AbdH in healthy youth. Ethical approval was granted by the South East Technological University Carlow Research Ethics Committee (Approval No: 284). Written informed consent was obtained from all participants and their legal guardians. Demographic information, height, and body mass were recorded at the outset of testing using a stadiometer and scales (213 and 875, Seca Corp., Hamburg, Germany). Participants comprised a convenience sample of *n* = 27 adolescents (14 males, 13 females) aged 11–17 years, who were free from lower‐limb injury or disability at the time of testing. The inter‐device association and inter‐rater reliability subgroup included *n* = 12 participants (5 males, 7 females), with a mean height of 168.2 ± 14.7 cm, body mass of 58.8 ± 16.2 kg, and age of 14.4 ± 2.5 years. The inter‐session reliability subgroup consisted of *n* = 15 participants (9 males, 6 females), with a mean height of 163.5 ± 12.4 cm, body mass of 56.4 ± 8.3 kg, and age of 13.9 ± 1.9 years.

### 2.2 Ultrasound Imaging

For the inter‐device association, two US devices were used to capture cross‐sectional images of the AbdH. The first device was a portable, wireless handheld linear scanner (5–15 MHz, L15HD, Clarius, Burnaby, BC, Canada) paired with an iPad (Pro version, second Generation, Apple, California, USA) and the second was a conventional cart‐based device equipped with a 3–16 MHz linear transducer (HS40, Samsung Healthcare, UK). The portable device provided a musculoskeletal foot/ankle preset, which was proprietary and non‐adjustable; however, for the cart‐based device, the gain, frequency, and focus were manually adjusted for each participant, as necessary, to optimise image quality. Images were analysed by the respective assessors who acquired them. Therefore, the reliability analyses reflected variability associated with both image acquisition and image analysis.

Data were collected across one or two sessions, depending on the reliability analysis. For the inter‐device association, Assessor 1 captured a single image of the AbdH from the left foot of each participant using both US devices within the same session. For inter‐rater reliability, Assessors 1 and 2 each captured three images from the same foot using a portable device during the same session. The measurement side (left or right) was randomly assigned using a coin toss. Each image was measured, and the mean CSA of the three measurements was used for the inter‐rater reliability analysis.

For inter‐session reliability, data collection was repeated across two sessions separated by 7 days, assuming foot muscle morphology remained stable over this period. The measurement side (left or right) was randomly assigned on day one using a coin toss, and the same foot was assessed at both sessions. Assessor 2 captured three images of the AbdH CSA from the same foot at each session, with the time of day standardised between sessions. Each image was measured, and the mean CSA of the three measurements from each session was used for the inter‐session reliability analysis. Assessor 1 had more than 5 years of musculoskeletal US experience, while Assessor 2 had 1 year of experience.

Participants lay supine with the hip slightly externally rotated and the ankle maintained at approximately 90° [19]. The assessor identified the AbdH by palpating the anterior aspect of the medial malleolus and marking a line distally (Figure 1) [19]. A gel pad (Aquaflex, Fairfield, USA) was placed between the US probe and the skin, without additional US gel, to minimise compression and preserve muscle architecture. The probe was positioned over the marked line and held perpendicular to the muscle border to optimise image quality. All images were coded, annotated, and stored following each session. CSA of the AbdH was quantified using digital callipers within the Clarius (portable) and Samsung (cart‐based) software, with the muscle border manually traced (Figure 2).

**FIGURE 1:**
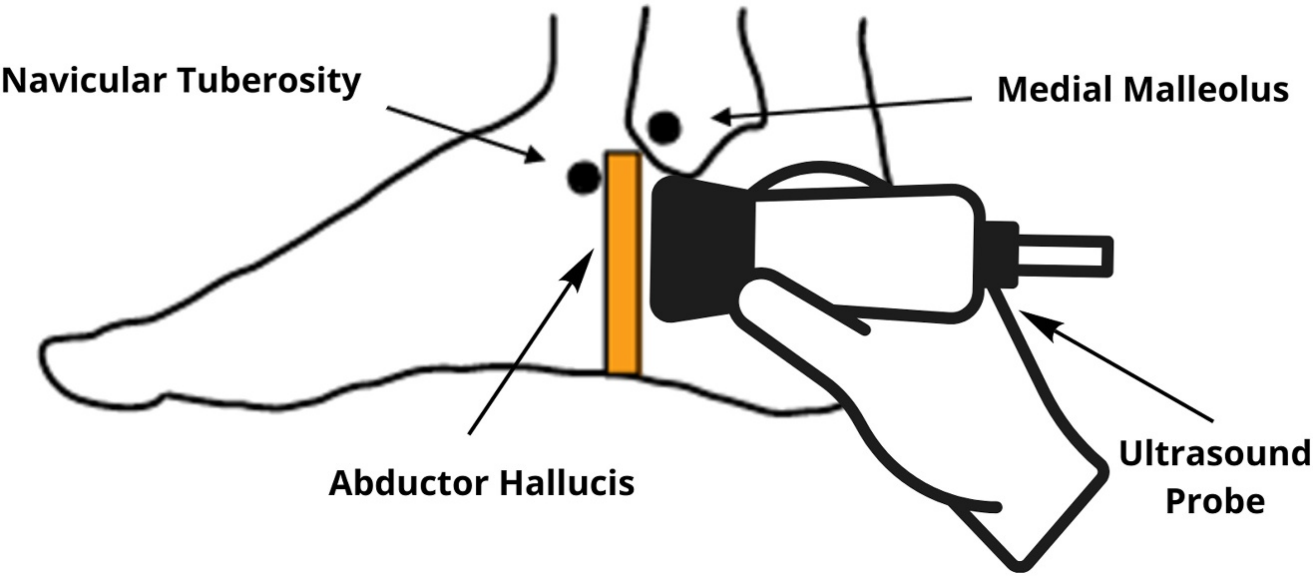
Schematic representation of anatomical landmarks used to standardise probe placement during scanning.

**FIGURE 2:**
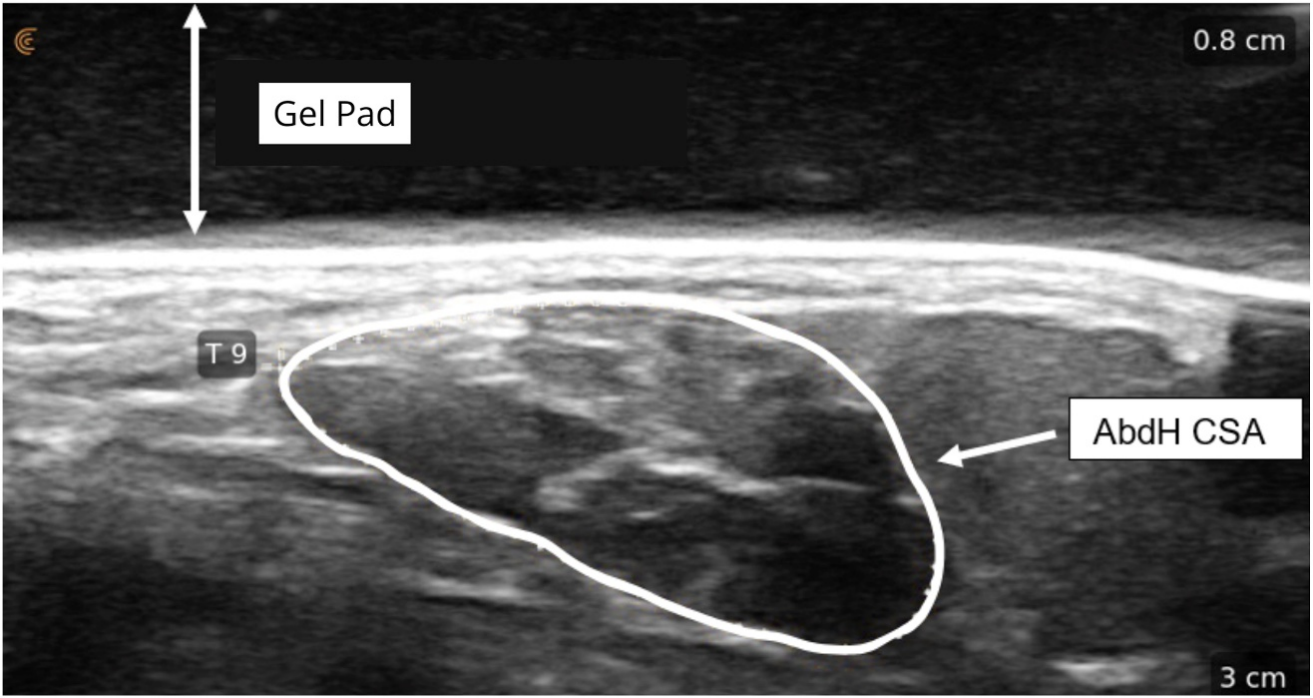
Ultrasound image of the abductor hallucis cross‐sectional area captured on the portable device.

### 2.3 Statistical Analysis

Inter‐rater and inter‐session reliability were assessed using a two‐way mixed‐effects, absolute‐agreement intraclass correlation coefficient for the mean of *k* measurements (ICC(3, *k*)), where *k* = 3. The mean of three measurements per assessor was used for all reliability analyses [18, 19]. Reliability was interpreted as moderate (ICC = 0.50–0.75), good (ICC = 0.75–0.90), and excellent (ICC > 0.90) [24].

All statistical tests were two‐tailed, with statistical significance set at *p* < 0.05. For inter‐device association, a single measurement from the left foot was used to perform Pearson's correlation, linear regression analyses, and systematic differences between devices were evaluated using a paired‐sample *t*‐test.

The standard error of measurement (SEM) was calculated as

$$SEM=SD\times \sqrt{1-ICC}$$

## 3 Results

Descriptive measurements of AbdH CSA, SEM values, and reliability statistics are presented in Table 1.

**TABLE 1:** Abductor hallucis (AbdH) cross‐sectional area outcomes.

Analysis	Comparison	Mean ± SD (cm^2^)	Mean ± SD (cm^2^)	Reliability	95% CI	SEM (cm^2^)
Inter‐device association (*n* = 12)	Portable versus cart‐based ultrasound	1.86 ± 0.40	2.05 ± 0.38	*r* = 0.938	—	—
Inter‐rater reliability (*n* = 12)	Assessor 1 versus assessor 2	1.83 ± 0.37	1.84 ± 0.43	ICC(3, 3) = 0.934	0.786–0.980	0.039
Intra‐rater reliability (*n* = 15)	Day 1 versus day 2	1.95 ± 0.42	1.94 ± 0.39	ICC(3, 3) = 0.969	0.911–0.989	0.013

Abbreviations: CI, confidence interval; ICC, intraclass correlation coefficient; SD, standard deviation; SEM, standard error of measurement.

### 3.1 Inter‐Device Association

A strong, statistically significant positive correlation was observed between the portable and cart‐based US devices (*r* = 0.938, *p* < 0.001). Mean AbdH CSA was 1.86 ± 0.40 cm^2^ for the portable device and 2.05 ± 0.38 cm^2^ for the cart‐based device. CSA was significantly higher for the cart‐based device (mean difference = 0.19 cm^2^, *p* = 0.002).

### 3.2 Inter‐Rater Reliability

No significant difference was observed between assessors (paired *t*‐test, *p* = 0.823). Inter‐rater reliability for AbdH CSA was excellent (ICC(3, 3) = 0.934; 95% CI: 0.786–0.980) (Table 1).

Bland–Altman analysis (Figure 3A) demonstrated a mean bias of −0.01 cm^2^, with 95% limits of agreement ranging from −0.31 to 0.29 cm^2^. The SEM was 0.039 cm^2^.

**FIGURE 3:**
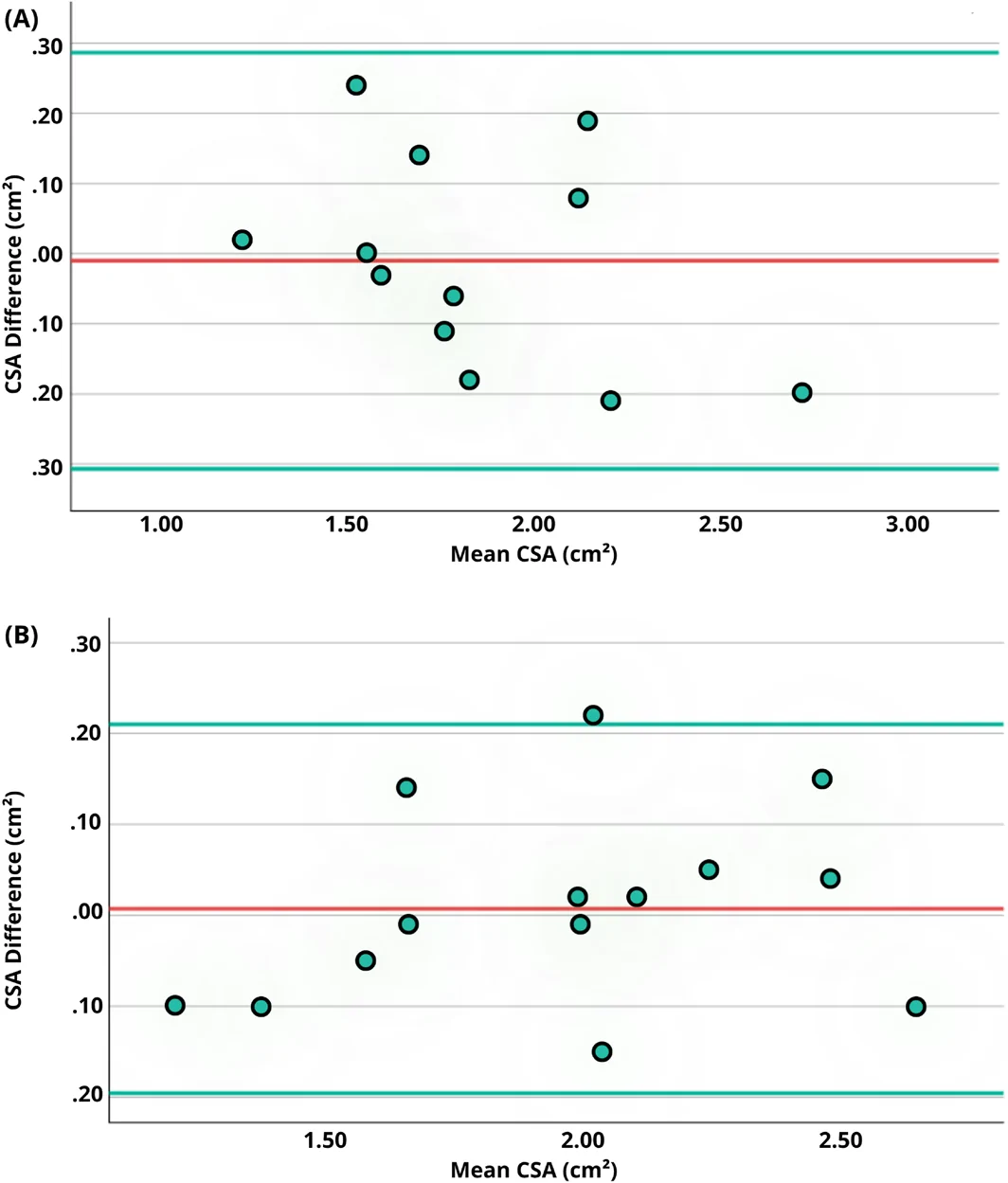
Bland‐Altman Plot for the (A) inter‐rater and (B) inter‐session reliability of measuring AbdH cross‐sectional area with the portable device.

### 3.3 Inter‐Session Reliability

No significant difference was observed between sessions (paired *t*‐test, *p* = 0.788). Inter‐session reliability for AbdH CSA was excellent (ICC (3, 3) = 0.969; 95% CI: 0.911–0.989) (Table 1).

Bland–Altman analysis (Figure 3B) demonstrated a mean bias of 0.007 cm^2^, with 95% limits of agreement ranging from −0.196 to 0.21 cm^2^. The SEM was 0.013 cm^2^.

## 4 Discussion

This study is the first to examine the inter‐device association, inter‐rater reliability, and intra‐rater inter‐session reliability of portable US for measuring the CSA of the AbdH in healthy youth. The results indicate that portable US provides reliable measurements in this population, with excellent inter‐rater and inter‐session reliability and a strong positive correlation with a conventional cart‐based system.

The present study demonstrated a strong positive correlation between the portable and cart‐based US devices (*r* = 0.938), indicating that both systems rank individuals similarly in terms of AbdH CSA. However, mean CSA values were significantly higher for the cart‐based device (mean difference = 0.19 cm^2^, *p* = 0.002).

This may reflect differences in image optimisation capabilities, such as adjustable gain, frequency, and focus, which may enhance muscle boundary definition in cart‐based systems. Differences in image scaling and measurement implementation between the two systems may also contribute to differences in CSA estimates. In the present study, CSA was measured using each device's native software, meaning that the measurement process was not standardised across systems. A common approach is to export US images and perform manual, semi‐automated, or automated measurements using the same external software, thereby standardising the measurement workflow across devices. However, determining whether this approach reduces inter‐device differences was beyond the scope of the present study and represents an avenue for future research in foot muscle imaging.

Previous research examining multiple US systems for IFM assessment is limited. Hing et al. [18] evaluated measurements obtained from both portable and cart‐based devices, but focused on intra‐tester reliability within each system rather than agreement between devices. Similarly, Willemse et al. [23] reported excellent reliability using a portable system, but did not assess inter‐device association. As such, the current findings provide preliminary insight into the relationship between measurements obtained from different US systems.

Inter‐rater reliability for measuring the AbdH CSA in youths was excellent (ICC (3, 3) = 0.934). This is consistent with previous studies by Crofts et al. [25] and Battaglia et al. [26] who reported excellent inter‐rater reliability (ICC = 0.98 and 0.95, respectively) for measuring AbdH CSA in healthy adult populations. The present study extends these findings to a youth population, indicating that portable US can be used consistently between assessors in this group. Notably, excellent reliability was observed despite a difference in assessor experience, which has been shown to influence US‐derived measurements in other tissues [27]. The high inter‐rater reliability observed in this study may reflect the use of a standardised imaging protocol, as well as the characteristics of the AbdH in a youth population. With ageing, adipose tissue tends to accumulate within the muscles, which can reduce the visibility of muscle borders [28]. In contrast, the AbdH in younger individuals may present with clearer anatomical boundaries, potentially reducing operator‐dependent variability. These findings support the use of US for reliable assessment of AbdH morphology in youth populations and may facilitate future research examining foot development across childhood.

Three previous studies have investigated US inter‐session reliability to measure the AbdH's CSA in healthy adults, with between‐session reliability reported as good [29] to excellent [19, 29, 30]. The findings of the present study (ICC (3, 3) = 0.969) are consistent with the literature, indicating that this measurement protocol can be applied reliably in a youth population using portable US. Comparable results have also been reported in clinical populations, including individuals with hallux valgus and pes planus, where between‐session reliability was excellent (ICC = 0.96–0.97) [5, 9]. The low SEM observed in the current study further supports the precision of repeated measurements and suggests minimal measurement error. This is particularly relevant for longitudinal or developmental studies, where detecting true physiological changes requires a stable and repeatable measurement approach.

Some limitations should be considered when interpreting the results of this study. The findings of this study are limited to the AbdH, one of the largest and easily identifiable IFMs; thus, whether similar results would be found in smaller foot muscles remains unknown. While previous, similar studies have used comparable sample sizes [31], our sample sizes for inter‐device association, inter‐rater reliability, and inter‐session reliability were modest, which is reflected in the width of the confidence intervals, particularly for inter‐rater reliability. For the inter‐device analysis, only a single measurement was obtained per device due to logistical constraints with the youth cohort. Previous work has suggested that multiple measurements may reduce measurement error when comparing US systems [18]. In addition, although the inter‐device analysis assessed association and mean differences, agreement between devices was not formally assessed. Therefore, conclusions regarding interchangeability cannot be made. Finally, the findings are limited to a cohort of healthy youths, which may restrict generalisability to other age groups or clinical populations. Future research should aim to replicate these findings across different populations and further evaluate the association of US systems.

## 5 Conclusion

The present study demonstrates that portable US provides good‐to‐excellent reliability for measuring AbdH CSA in a youth population. These findings indicate that AbdH CSA can be assessed consistently using portable US across and within assessors. A strong positive association was observed between portable and cart‐based US devices; however, these findings relate to association rather than interchangeability. Portable US may therefore provide a practical approach for assessing AbdH morphology in field‐based settings. Future research should aim to replicate these findings across different age groups and clinical populations, and further evaluate the association of US systems to support the development of normative data.

## Author Contributions

**Maisie Squibb:** conceptualization, data curation, formal analysis, methodology, investigation, writing original draft, resources, visualization, project administration. **Dean Monks:** writing, reviewing, editing. **Christopher D. Nulty:** writing, reviewing and editing. **Kelly Sheerin:** conceptualization, methodology, formal analysis, writing, reviewing and editing **Peter Francis:** supervision, resources, funding acquisition, writing, reviewing and editing.

## Funding

The authors have nothing to report.

## Ethics Statement

The ethical committee of South East Technological University Carlow provided ethical approval for this project (284).

## Consent

Written informed consent was obtained from all subjects and their legal guardians.

## Conflicts of Interest

The lead author M.S. and an additional co‐author D.M. received funding toward the completion of their PhD from the minimalist footwear company Vivobarefoot. P.F. receives funding for research projects from the minimalist footwear company Vivobarefoot. P.F. advises the Live Barefoot Foundation on its scientific strategy and the accuracy of its outreach activities; he receives an honorarium for this work. The company had no involvement in the study design, data collection, analysis and interpretation of data, or the writing of the report.

## Data Availability

The data that support the findings of this study are available from the corresponding author upon reasonable request.
